# Role of frequency modulation radio in disaster communication: A case study of Pakistan earthquake

**DOI:** 10.4102/jamba.v13i1.1047

**Published:** 2021-11-30

**Authors:** Syed I. Rahman, Nauman Sial, Saniya Moazzam

**Affiliations:** 1 Department of Media and Communication Studies, Faculty of Social Sciences, International Islamic University, Islamabad, Pakistan; 2 Centre for Media and Communication Studies, University of Gujrat, Gujrat, Pakistan

**Keywords:** Pakistan earthquake, disaster communication, FM radio, community media, social responsibility

## Abstract

Pakistan suffered from a massive earthquake in October 2005 that caused the deaths of more than 87 000 people. As a result of this calamity, around 3.5 million affected people had no access to information. In these scenarios, community media became an important catalyst. In developing countries, radio had proved far more accessible and useful than any other medium. But because of this natural hazard, local media also suffered heavily as dozens of journalists died and media houses and press clubs were destroyed. The current study attempted to explore the role of frequency modulation (FM) radio stations working in the earthquake hit areas in Pakistan. These stations were temporarily setup to inform the victims about the rehabilitation and reconstruction plans of the agencies involved. The data has been collected qualitatively through five focus group discussions which were conducted in the earthquake affected areas. Twelve in-depth interviews were also conducted for this purpose with FM stations personnel. The results revealed that the FM radio stations played a very important role in the rehabilitation phase by providing vital information to the victims, relief agencies and government. Lifesaving information like weather updates, precautionary measures in the tents, public service announcements and encouraging messages provided some hope to the victims to restart a normal life, and also motivated the students to restart their studies in makeshift schools.These FM networks became the voice of the affected people and helped a lot in bridging the communication gaps between the affected, relief agencies and government, and also ensured citizens’ participation in decision-making processes.

## Introduction

On 08 October 2005, a 7.6 magnitude earthquake struck Pakistan that caused enormous destruction (Sajid 2019). The earthquake affected an area of almost 30 000 square kilometers including the North West Frontier Province (nowadays Khyber Pakhtunkhwa, [KPK]) and federally administered state of Azad Jammu and Kashmir (AJK). As a result of this natural hazard, more than 87 000 people had lost their lives and around 4 million people were left homeless (Sajid 2019; Shabbir 2015). In terms of linkages amongst communications technology and numerous kinds of hazards, radio and television can transmit warnings and protection information during the earthquake disaster. Both radio and television might broadcast early warnings and evacuation information and, can also increase public awareness regarding the risks and responses (Rattien 1990). In the scenarios of calamity, community media becomes an important catalyst. Community media usually refers to, ‘a site that not only indicates considerable audience activity but vividly demonstrates tangible audience power’ and ‘underscore the creativity, pragmatism, and resourcefulness of local populations in their struggle to control media production and distribution’ (Howley 2005:3). Amongst different types of community media, in developing countries, it is the radio that proves far more accessible and useful than any other medium (Dwivedi 2010; Hibino & Shaw 2014):

A community radio station is characterized by its ownership and programming and the community it is authorize to serve. Its programming should be based on community access and participation and should reflect the special interests and needs of the listenership it is licensed to serve. (Fraser & Estrada 2001:4)

Community radio can play the role of promoter for participatory, accountable and transparent governance in a society affected by natural hazard and it can also create enormous opportunities for regaining the mental and physical losses. Radio can be the main source of information especially after the earthquake when electricity power goes out. Because of these advantages, radio becomes a very important medium during disasters (Dwivedi 2010; Hibino & Shaw 2014; Puzon-Diopenes & Murshed 2006). Radio Pakistan came into being on the Independence Day of Pakistan, that is, 14 August 1947. There were only two radio stations at that time (in the cities of Lahore and Peshawar). Since its creation, Radio Pakistan has been owned by the government. In 1972, through an Act of Parliament, it was renamed as Pakistan Broadcasting Corporation (PBC). During the 1990s, radio was the prime source of entertainment for almost half of the population. By 1997, state-owned and government controlled PBC had 22 radio stations in all the major cities of Pakistan. Pakistan Broadcasting Corporation is the only medium in Pakistan that broadcasted programmes in 20 different languages and dialects on daily basis (Ahmad 1991; Gilani & Zuberi 1993; Jabbar 1997). In Pakistan, almost 70% of population live in rural areas (Chaudhry 2013), where the literacy rate is 49% (Haq 2017). So because of low literacy rate and poverty in the rural areas, people have little access to newspapers (Chaudhry & Rahman 2009; Rehman, Jingdong & Hussain 2015), whilst the state-run Pakistan Television (PTV) do not cover all regions of the country owing to lack of booster towers (BBG 2014; Yusuf 2013). As a result of the 2005 earthquake, the local media also suffered heavily as dozens of journalists died and newspaper offices, broadcast houses and press clubs were totally or partially destroyed. The state-run Kashmir Radio and TV were also destroyed and around 40 of its staff died in this event. The calamity was on the front pages in world media, but 3.5 million affected people of the earthquake had no access to information about how to get help and relief especially in AJK (Rehmat 2006).

Soon after the earthquake, Internews (an international organisation), and Association of Independent Radio (AIR) of Pakistan appealed to Pakistan Electronic Media Regulatory Authority (PEMRA) for grant of emergency temporary broadcast licenses in the areas struck by the earthquake. In this scenario regarding information access, setting up of emergency radio station was the only way out to cater to the needs of the people who were in dire need of information about relief activities. So PEMRA granted temporary licenses to private communication organisations for setting up relief and emergency frequency modulation (FM) radio stations on non-commercial basis in the quake-hit regions (ReliefWeb 2005). For this purpose, Internews donated 10 000 radio sets which were distributed by United Nations High Commission for Refugees (UNHCR) amongst the people of earthquake hit areas (Dawn 2006). After the earthquake, private FM radio stations were a new phenomenon in the affected areas and for the first time local media experienced the coverage of such a huge disaster in the history of Pakistan. The current study attempts to explore the role of FM radio stations working in the earthquake hit areas of Pakistan which were temporarily set up to inform the earthquake victims about the rehabilitation and reconstruction plans of the agencies involved.

### Literature review

Most communication scholars are of the view that media could be used for development in developing countries (Thussu 2000). Similarly, disaster hit areas also require development in the form of reconstruction and rehabilitation (Isayama & Shaw 2014). Rooney (2012:38) argues that, ‘people need information as much as water, food, medicine or shelter. Information can save lives, livelihoods and resources. Information bestows power’. In any tragedy, media has a crucial role to play. The coverage of the effects of calamity to the world can contribute to human understanding and universal sympathy. At the same time, providing information to the affected people might enable them to better understand the happenings around them, and that can lead to empowerment. It is also the duty of media to conduct investigative research, and to trace the disbursement of aid to help as well as to ensure responsible and transparent accountability (Dwivedi 2010; Nayak 2012; Puzon-Diopenes & Murshed 2006; Rattien 1990). Quarantelli (1996) said that disaster preparedness planning amongst local mass media organisations is very limited and generally of poor quality. Little attention is given to emergency and disaster planning in most media organisations.

Melkote and Kandat (2001) pointed out regarding development support communication (DSC) that there is a need to create an environment in which psychologically and economically disempowered individuals can discuss with experts and policymakers on an equal footing. However, the concept of development communication (DC) advocates the top-down approach. In this type of communication, the masses are not allowed to make decisions, and it does not help to make necessary social change. This underlines the fact that if the audience feels that the message is imposed upon them from outside, it is highly unlikely that they would be receptive to it. For media interventions to be successful or any programmes to empower communities, there must be a local identity with specific achievable targets, even in times of natural hazards. Fisher (2001) argued that if development is to be effective, local goals must tie the mass media in projects to traditional communication channels. A multimedia approach coupled with an innovative educational strategy seemed to be most appropriate if adoption of an idea is to take place. Development problems cannot be solved by just throwing information at the receivers.

In the milieu of disaster communication, portable communication through satellite could deliver reliable, immediate, and personalised communications for disaster respondents, irrespective of the severity and magnitude of the surrounding damage (Garshnek & Burkle 2007). Sreedher (2005) argued that community radio can be effective during a disaster only if it has access to authentic information. It cannot operate in isolation and must work in consonance with national media at such times. Amor and Murria (2005) elaborated on the complications in the relationship between the disaster and media. They stated that communication is one of the most significant and pressing features in a disaster risk reduction programme. Knowing what to communicate, to whom, by whom, how, when and where are of utmost importance for a successful disaster risk reduction programme by helping to overcome the worldwide barriers of ignorance, apathy, organisational and disciplinary boundaries, and lack of political will in every community and to build a global culture of community resilience to natural and technological hazards. The way the media report and comment on disasters will, in good measure, shape the way in which the community react in the face of a disaster, before, during and after its occurrence.

Puzon-Diopenes and Murshed (2006) discussed that during the post-disaster phase, media can appeal for assistance from all parties, to communicate about rehabilitation and reconstruction plans, to encourage survivor participation in recovery, and to influence for integrating risk reduction and prevention. Scanlon (2007) explained that mass media play an important role in various aspects of crises and disasters. The participation of media is critical before, during and after such incidents. As for effective warning, media can be the glue that binds citizens in certain instances, and could be the only most vital source of public information in the wake of a disaster. However, media is also responsible for several misconceptions that exist regarding disaster. These fallacies could also lead to judgement errors when disaster hits. So, media must be monitored and handled with care, and media can also be critical in putting down rumours.

### Theoretical framework

The theory of social responsibility has been used as a theoretical framework for this study. Social responsibility is an ethical theory of the press. The theory states that the press has a responsibility to preserve democracy by properly informing the public and by responding to society’s needs and interests (Severin & Tankard 2014). The theory originated after the Hutchins Commission report on A Free and Responsible Press in 1947. According to this theory, the press should present truthful and equal side of every issue and if the press is not fulfilling its functions, then a regulatory authority must enforce it (Christians et al. 2009; Severin & Tankard 2014):

The power and near monopoly position of the media impose on them an obligation to be socially responsible, to see that all sides are fairly presented and that the public has enough information to decide; and that if the media do not take on themselves such responsibility it may be necessary for some other agency of the public to enforce it. (Siebert, Peterson & Schramm 1956:5)

McQuail (2010) summarised the basic principles of theory as that media should accept and fulfil certain obligations to society. These obligations are mainly to be met by setting high or professional standards of informativeness, truth, accuracy, objectivity and balance. Also, media as a whole should be pluralist and reflect the diversity of their society, giving access to various points of view and rights of reply (Ravi 2012).

## Methodology

The current study proposed two research questions, that is, what content was provided by the FM stations to the listeners? And how was the information received and used by relief organisations and affected people (survivors and general public)? For study purpose, the two forms of qualitative data collection methods, that is, focus group discussion (FGD) and in-depth interviews have been used as a research design. Focus group discussion is a very useful method for eliciting information from a group of people after a thorough discussion on a specific subject. During the discussion, besides the main questions, other aspects of the topic also highlight which further strengthen the findings. Every individual approach used to be different towards the issue under discussion which not only provides insight to the researcher but also draws special attention to the neglected parts. The selected participants for the focus groups must be involved in a particular situation, for example, they must have seen a movie or have heard a radio programme (Deacon et al. 1999; Hennink 2013). Likewise, an intensive or in-depth interview is a specific form of interaction between two people, the researcher, and the informant. They both act in relation to each other as well as influence each other. During the interview, the researcher asks questions from the interview guide and follows the chosen topics until the interview is over. The purpose of in-depth interview is to understand the meaning of the subject’s special life-world, examining both what is said and in the way it is said (Kumar 2005; Minichiello, Aroni & Hays 2008; Wimmer & Dominick 2013).

The participants of focus groups were selected through purposive sampling. A total of five FGDs were conducted in the earthquake affected areas. All the participants were amongst the quake hit areas, and were frequent listeners of one or more programmes of emergency FM radio stations. In the first group, seven members participated whilst in the remaining four groups, six members each took part in discussion. All groups had a mix of participants with a varied level of education and work backgrounds, including highly educated professionals such as doctors, teachers, journalists, non-governmental organisation and social workers, politician, businessman, government employees, lawyer, head of relief association, members of religious groups, hotel manager, nurses, housewives, and students. The other participants were shopkeeper, contractor, farmer, and supplier. Thus from the total of 31 members, 21 were males and 10 participants were females. The age of the members ranged from 21 to 50 years. In all the five FGDs, the questions regarding the type of information that was provided by the FM radio stations to the earthquake affected people and to the relief organisations were discussed. All the FGDs were conducted from 20 March to 20 April 2006, and were conducted in the national language of Pakistan, that is, Urdu. In addition, a total of 12 interviewees were selected through purposive sampling. The interviewees were working for eight different private owned FM radio stations based in the earthquake hit areas. Apart from two, all the FM stations started functioning in late October 2005. Out of 12 interviewees, nine were station managers and three were radio reporters. All the interviewees were non-local except for two station managers. Separate sessions were conducted for each interview. The interviewees were experts in their field but they had no experience of working in the disaster hit areas. It took 30 min – 45 min to complete a single session.

## Results

The FM radio stations broadcasted a variety of programmes in local languages which were based on information and entertainment. Besides these programmes, back-to-back music was also an important segment of these stations. The World Health Organization (WHO), Save the Children, Oxfam and local health departments initiated a massive vaccination drive to control the possible outbreak of the epidemic diseases. An interviewee said:

‘WHO wanted to arrange a medical camp about ‘scabies: a transmissible skin infection’ but they had a very little time to inform the people. They contacted us and we on hourly basis made public service announcements (PSAs) which ensured attendance in large numbers.’ (Station manager, FM Power 99)

A doctor who worked with WHO and took part in the FGD commented the following:

‘During tsunami, the concerned agencies had failed to control the outbreak of epidemic diseases, but timely information about the urgent need of vaccination against the diseases was promptly conveyed by the media especially the radio, and we succeeded in controlling all the diseases which usually spread after the disaster.’ (Doctor, FGD)

Focus group discussion participants and respondent interviewees agreed that people living in valleys and small villages on the hilltop had no idea about the vaccination, and they were informed by the FM station programmes that the vaccination will save them from further loss.

Children, elderly people and pregnant women in the quake zone were in dire need of precautionary measures. A housewife, who participated in the FGD, discussed that before the disaster, they could usually visit hospitals or clinics to consult doctors but the earthquake had destroyed the whole infrastructure, and the handling of injured children and women was a daunting task. She stated the following:

‘The continuous aftershocks multiplied the worries of pregnant women and many of them due to ignorance or fear resorted to abortion. Programs like *Online Clinic* invited lady doctors who guided the pregnant women about precautionary measures.’ (Housewife, FGD)

She further said that paediatrics in live programmes informed the parents about safety measures for infants in such unfavourable circumstances. Another FGD participant expressed:

‘I received treatment for my multiple injuries in Abbottabad Hospital and was sent back home [*tent*] due to heavy rush in the hospital. Lack of resources and land-sliding confined me to my tent so I got all the tips about healthy life from radio.’ (Businessman, FGD)

Another who participated in focus group said:

‘The radio through its motivational programs literally provided a new life to the survivors. The women and children living in camps relied heavily on the radio for guidelines about safe and healthy living.’ (Social worker, FGD)

An interviewee who was a reporter said:

‘We were very particular about the outbreak of water-borne diseases in a certain village. Our reporters visited the area and recorded the comments of the victims.’ (Reporter, FM Buraq, Abbottabad)

She added that this practice also alerted the agencies who were responsible for the health of children. Almost all the members of the group discussions agreed that FM radio stations working in earthquake zone was very important as information providers about safety measures.

The psychiatric doctors and physiotherapists both from local and foreign visited the hospitals where the sufferers were receiving treatment. They guided the patients on how to start a new life. An interviewee stated:

‘We interviewed many doctors and they provided very useful information to the victims about recovery from trauma. On the insistence of audience, we often rebroadcasted these programs.’ (Station manager, FM Buraq, Abbottabad)

Another interviewee said:

‘During our field visits, we asked the parents about the problems of their children, and then discussed those problems with the doctors in live programs and this interaction definitely provided a solution to their problems.’ (Reporter, FM Power 99, Bagh)

To provide basic amenities to the camp residents was a challenge for relief organisations and to keep the tent villages’ environment clean was also important:

‘United Nations International Children’s Emergency Fund (UNICEF) arranged a ‘cleanliness week’ in the affected areas and during this whole week, the radio stations in their programs interviewed experts and officials of the district administration to inform the people about importance of cleanliness. In live programs, local people also participated and took instructions from the experts about cleanliness in their tents.’ (Journalist, FGD)

It is pertinent to mention here that relief agencies along with other relief goods also provided bottles of mineral water. But, rumours spread amongst the victims that bottled water was not fit for drinking and consuming. On this issue, an interviewee who was a reporter discussed that radio programmes convinced them to use the mineral water for drinking purposes.

An FGD participant said that besides other problems, helping the people against cold was a very serious challenge for all the concerned agencies and the spread of weather-related diseases like fever, flu, pneumonia was also feared. He added:

‘All our advice and planning were on air from the FM stations and in this connection, I was confident to say that these messages saved the lives of many people who were living in unreachable areas of the earthquake zone.’ (Doctor, FGD)

Thousands of tents were set up to accommodate the homeless survivors but they were not used to living in tents. Several deaths were reported from burning of tents because of cooking inside the tents and the majority of victims were children:

‘Burning of tents along with its residents was alarmingly increased during the month of December. With the help of experts of camp management, we prepared PSAs to educate the people. This drive educated the people and the incidents were reduced.’ (Reporter, FGD)

It is interesting to mention that the people living in tents used to rub kerosene oil on the roof of tents to protect themselves from mosquitoes’ bite. But, this resulted in the burning of tents. On this matter, an interviewee discussed that radio reporters investigated the tent burning incidents and informed the people about how to protect themselves against mosquitoes. He added that radio programmes also advised the relief agencies to arrange insecticide in the tents.

The government of Pakistan invited seismic experts from Japan, Turkey and Korea to prepare a report on the nature of earthquake and its future prospects. The report declared some areas as red zone which were not fit for construction because of the possibility of earthquakes in future. The residents of the red zone were not happy with the decision. But, the radio programmes invited the responsible government officials who further elaborated the report and ensured the residents that they will be provided alternate lands for construction of houses. An interviewee said that people out of sheer disappointment came on the roads as a protest against the zoning report. With the passage of time, all this confusion was cleared by the radio live shows and other informative programmes about the issue. In addition, aftershocks multiplied the miseries of the survivors and a rumour was circulating that another powerful earthquake would hit the area. To counter this rumour, the government used the FM radio, and the weather experts in the radio programme calmed down the people by giving the examples of Japan where earthquake and aftershock is a routine activity.

Frequency modulation stations also updated the listeners about the activities of relief organisations:

‘We interviewed the concerned heads of the organizations who provided information to the listeners about their line of action. The survivors were in dire need of corrugated sheets so we informed the people about the organization responsible for its distribution and the procedure.’ (Reporter, FM Power 99, Abbottabad)

‘Some of the people of affected areas moved to their relatives’ house in other cities. This deprived them of compensation amount and they also could not take part in the reconstruction process.’ (Social worker, FGD)

The radio stations also updated the listeners about the compensation amount. The radio staff consulted with the concerned officials like head of the district, and government based Earthquake Rehabilitation and Reconstruction Authority (ERRA) officials who provided detailed information about the compensation process. Just like ERRA, the affected people had also formed alliances to convey their reservation to the officials about the government’s planning. For example, in Balakot, the Balakot Earthquake Relief and Reconstruction Association (BERRA) was in touch with FM Mast Balakot, Buraq Abbottabad, and Power 99 for conveying the people’s aspirations to the government:

‘The government had announced alternate townships for the survivors of Balakot but the proposed planning for townships was faulty as size of houses was not according to the people wishes. The people reservations were conveyed to the government by media especially by the FM radio stations.’ (President of BERRA, FGD)

An interviewee said:

‘Our reporters were invited for the field operation. The reporters went with UN officials in helicopters to the inaccessible areas and they brought interesting reports. This activity mobilized more relief goods to those areas.’ (Station manager, Power 99, Abbottabad)

In Muzaffarabad, 20 volunteers from various European countries were working with International Organization for Migration (IOM) to repair the tents damaged by rains or winds. On every rainy day, IOM officials requested the FM stations to inform the people about this facility. Another interviewee who worked as station manager said, ‘The IOM official told me that due to your announcements (PSAs), we received more than 80 calls on every rainy day for help from the people’. He added, ‘Weather update was very important segment and we were in constant touch with meteorological office in Islamabad’ (capital of Pakistan). A reporter and an interviewee commented that the dwellers of tent villages were heavily dependent on weather updates from the radio. They could arrange the outdoor activities according to the weather like washing of clothes and cooking food in the open sky.

An FGD participant, who worked with a relief agency, discussed that their workers used to listen radio weather updates before starting any operations in the hilly areas. She said that affected people also adjusted their schedule according to weather updates. An interviewee stated:

‘In our talk shows and other live programs, we highlighted the importance of protection of environment which not only sensitized the people in this hour of trial but also will have positive effects in future.’ (Station manager, Power 99, Bagh)

He further added that because of wide range of coverage, the PSAs and discussions made a great impression. The focus group participants and individual interviewees were unanimous that the FM updates about weather, government and relief agencies’ planning were very important for the people of earthquake hit areas, and the PSAs were also guiding the people in right directions.

The earthquake destroyed schools and colleges, and around 18 000 students had died. Those who survived were in depressed condition. The reconstruction of schools and colleges was a lengthy process but organisations like United Nations Development Programme (UNDP) and UNICEF opened schools in tents and also provided free books to the students. A lecturer by profession, who took part in focus group, said that these areas in terms of literacy were very backward. But, the earthquake destroyed schools and colleges, and the buildings that survived the shocks, had developed cracks. The makeshift schools were made available but attracting students was a big problem. The FM radio stations in their children specific programmes encouraged the kids to attend school and they were also informed that the tented schools could not collapse. The students of the makeshift schools were interviewed by the reporters who encouraged other children to restart their studies. The officials of various relief agencies arranged colourful events for the students to engage them in some positive activities. The radio reporters provided live coverage of these events and the young students’ comments attracted other children to the schools as well. The radio reporters also interviewed educators who guided the students on how to restart their studies in such circumstances. The FM stations in collaboration with UNICEF prepared interesting programmes for the listeners which encouraged the students to attend the schools:

‘My kids listened to *Bache Sab Se Ache* (transl. Children Best from All) with attention. It gave motivation to them to restart their studies and join the tented schools opened by the international organisations.’ (Farmer, FGD)

Almost all FGD participants appreciated the role of radio in motivating the students to restart their studies in the makeshift schools.

The FM stations also played a very important role in locating missing people. Relief agencies and social welfare departments had established information centres in various parts of the country. The radio reporters were in touch with these centres and provided details of those who were living in tents or were in hospitals for treatment. The radio stations provided a link between people and the centres. Several kidnapping incidents of the affected children were also reported in national and international press. The orphaned children were exposed to human trafficking. To overcome this problem, International Labour Organization (ILO) and IOM in collaboration with local authorities initiated a drive for the safety of homeless children. The FM stations interviewed the concerned officials who sensitised the people as how to check this menace. A teacher, who took part in FGD said, ‘We got information about our relatives from the broadcast of the FM radio station’. These stations also interviewed the people who were living in faraway camps and their relatives got clue from these programmes. Sometimes, the injured survivors were airlifted by the rescue agencies to big cities for treatment but their relatives were unaware about their locations. An interviewee said that, despite limited resources, FM stations through their various programmes provided information about missing people.

In any hour of trial it is perceived that every religion provides a solace and lessons of tolerance and passion to the affected people. All the stations allocated enough time for religious programmes which were appreciated by the public. An interviewee stated:

‘We broadcasted speeches of famous scholars which had soothing effects on the listeners. These programs also convinced the people that earthquake was not a punishment for the human being’s sins.’ (Station manager, Power 99, Muzaffarabad)

Every morning the FM stations took start from recitation of the Holy Quran and broadcasted recorded speeches (sermons) of religious scholars who inspired the audience spiritually. One FGD participant who was a student said, ‘The programs aired by the FM stations about our religion provided us consolation and peace of mind, and motivated us to start a new life’. The station manager of Power 99, Abbottabad discussed that the survivors especially the children and women living in tents listened to radio very much as they had lost other sources of entertainment. He added that the injured, who were advised by the doctors for bed rest, were fully involved in the radio programmes and actively participated by using their cell phones. The station manager of FM Power 99, Muzaffarabad stated, ‘In the beginning, we were concentrating on information but with the passage of time, we started receiving phone calls from listeners who were demanding music and songs’. An interviewee who worked as a reporter said that the radio stations increased the duration of entertainment based programmes because of public demand:

‘Our station received phone calls and even letters for requesting of songs and we instructed the DJs that during the entertainment programs they must also convey some persuasive messages for new life.’ (Station manager, Power 99, Bagh)

One FGD participant, a reporter by profession said that some land owners, in order to get more compensation amount and other benefits started evacuation of their tenants. In this connection, the officials during the interviews informed the tenants that they should not vacate the lands, and also warned the land owners about legal action. He added that after this report, the forceful evacuation was stopped. Also, many tent villages had been set up hurriedly and they lacked basic amenities like water and electricity. A reporter and an interviewee discussed that reporters highlighted the problems of tent villages and the concerned agencies provided electricity to the camps in a short time. An interviewee commented:

‘The government had announced free electricity in the affected areas for three months but the Water and Power Development Authority (WAPDA) sent electricity bills in some affected areas. The people complained to the radio through phone calls and letters. The reporters discussed this issue with WAPDA authorities and reminded the government announcement. They stopped sending bills to the affected areas.’ (Station manager, FM Buraq, Abbottabad)

He added that landslides and building collapses had disconnected telephone lines in the affected areas but the people continued receiving phone bills. The reporters informed the telecommunication officials who not only cancelled the previous bills but also repaired the lines. An interviewee, station manager of FM Mast, Balakot said that the sale of relief goods became common in the local markets of the affected and adjacent areas. He added that the stations staff highlighted the issue and also discussed it with the responsible officials. The police as a reaction established check post to control the flow of such goods and also started monitoring of local markets.

Another interviewee and station manager of FM Sachal, Rawlakot commented that the government through revenue officials started distribution of forms for compensation. The forms were free of cost but the officials started selling them. He said that the issue was highlighted by the stations and this practice was stopped immediately. Also, to obtain compensation, a Computerized National Identity Card (CNIC) had to be shown, but many people had lost their cards during the disaster. The station manager of Power 99, Bagh discussed that the reporters interviewed the people who complained about this problem and also contacted with National Database and Registration Authority (NADRA) officials. He added that right after 1 day, NADRA mobile team visited the affected areas and issued identity cards to the victims on emergency basis. The government gave the compensation amount in the form of cheques and to cash the cheque, a bank account was necessary. One FGD participant, a reporter by profession said that majority of the survivors were poor and they had no bank accounts, and they had to come from adjoining large towns for opening their bank accounts. He added that the issue was highlighted by the reporters and contacted the officials of the National Bank of Pakistan (NBP) who provided bank counters in small villages and towns. This facility saved the time and money of the affected people. The station manager of Power 99, Bagh also commented that the stations’ staff could not afford to highlight irregularities and faulty policies of the government at a high level as their main objective was to provide information to the affected people. However, the FM radio played the watchdog role in a very befitting manner as far as problems of the affected people were concerned.

The FM radio stations had been issued temporary licences only for 3 months but the government extended the licences for 3 more months whilst the government announced reconstruction plan in March 2006. The station manager of FM Power 99, Muzaffarabad said that FM stations’ role in the reconstruction was more important as all the relief agencies had chalked out awareness campaign for the public regarding the reconstruction plan. He added that the affected people were not clear about the reconstruction plan and they still needed more informative programmes. Various relief agencies started collaboration with the FM stations and prepared informative programmes regarding the reconstruction. The radio stations empowered the local people in terms of including their voices in the decision-making process:

‘They felt satisfaction that their voices were being heard by the decision-makers.’ (Station manager, Power 99, Bagh)

‘Our station was situated on hill-top but even then, people visited our office with applications for help. We told them that we could only broadcast their problems. So their problems were highlighted and resolved immediately.’ (Station manager, FM Mast, Balakot)

He further said that the radio fully bridged the gap between the relief agencies, government and survivors:

‘Norwegian Refugees Council (NRC) was holding camp management training and we informed the people to register them for the training. After few days, the concerned NRC officials thanked us for informing the people. All the participants got this information from radio.’ (Reporter, FM Power 99, Abbottabad)

An interviewee expressed that the radio staff also attended UN coordination meetings on a daily basis and they provided suggestions for expediting the relief work:

‘For updating the public, we gave coverage to these meetings and the UN staff also appreciated our suggestions.’ (Station manager, FM Buraq, Abbottabad)

‘We anxiously waited for our favourite programs and also participated in the programs by giving our feedback through phones.’ (Student, FGD)

The FGD participants agreed that the affected people felt a sense of boldness when they listened to their own voice on the radio. They could not imagine hearing their voice on the state-controlled radio. The local people also visited the radio stations to record their comments and suggestions about problems. Commenting on the concern about the increasing dependency of the survivors on aid, an interviewee expressed:

‘We literally prevented the people from begging. Through our programs, we requested them to stop waiting for aid and start a new life within their own resources.’ (Station manager, FM Sachal, Rawlakot)

‘We interviewed affected families who were reconstructing their houses. They persuaded other affected people to start a new life and to forget the past.’ (Reporter, FM Power 99, Abbottabad)

The government and welfare organisations had set up rehabilitation centres for widows of the affected areas but those living in tents and with distant relatives had no information about these centres:

‘We informed the widows about the centres and also guided them how to approach it. We also visited these centres and highlighted the facilities being offered to the widows so this attracted them.’ (Reporter, FM Buraq, Abbottabad)

Brook Hospital, a UK-based trust working for the welfare of livestock, established camps in the earthquake affected areas to treat the injured livestock, and to provide food and shelters for the survived animals:

‘We visited various villages to inform the people about our work, but there was no response as they were busy in removing debris of their houses and mourning their losses. We gave the schedule of our medical camps to local FM radio stations who broadcasted this in the form of PSAs and also convinced the people that livestock needed attention. The radio messages convinced the people for initiating contacts with our teams.’ (Veterinarian, FGD)

‘In terms of information, the PSAs were the most important item and we fully cooperated with relief agencies in preparing it.’ (Station manager, Power 99, Bagh)

The earthquake happened in the Islamic holy month of Ramadan, and at the end of this month, Eid-ul-Fitr, a religious festival used to be celebrated throughout the Muslim world. During Eid, local and international dignitaries and prominent social figures visited the earthquake hit areas and expressed solidarity with the victims. The radio programmes updated the listeners about the activities of these guests which encouraged them.

## Conclusion

The FM radio stations that worked in the earthquake hit areas of Pakistan played a very important role in the rehabilitation phase by providing vital information both to the victims and international relief agencies, and this role continued in the reconstruction phase. On 08 October 2006, 1 year after the earthquake, the UN published a report on its role in the earthquake hit areas which appreciated the positive role of media and especially of FM radio (Tan 2006). Also, an information access survey was conducted by Internews in the affected areas. The survey revealed that after a few weeks of the disaster, 23% affected people relied on radio for information. But in February 2006, this figure had gone up to 70% (Rehmat 2006). With regard to the theory of social responsibility, these FM radio stations properly informed the affected people and responded to the society’s needs and interests. Frequency modulation stations acted in a socially responsible manner by presenting all sides in a fair manner, and the victims had enough information to decide. These stations reflected the diversity of the affected society, and gave access to various points of view and rights of reply. Initially, PEMRA had issued 3 months license to these stations, but after realising its importance, the licenses were extended for another 3 months. The national and international television channels and print media played a very important role in informing people of the world about the disaster and destruction. Within Pakistan, FM radio stations became the voice of the affected people, and ensured a two-way communication that helped in bridging the communication gaps between the affected, relief agencies and government. The radio programmes targeted a wide range of audience such as opinion leaders, government officials, relief agencies and the victims. Besides becoming the victims’ voice, the FM radio also introduced new trends which could help in the development of the backward areas. Lifesaving information like weather updates, precautionary measures in the tents, PSAs and encouraging messages provided a ray of hope to the victims.

The FM radio also played a very crucial role in motivating the students in restarting their studies in the makeshift schools. The recovery of the victims from the trauma was a very daunting task but the FM radio involved the victims in their programmes and they were motivated to start a normal life. The FM stations ensured people participation in decision-making processes. In live call-in shows and other programmes, people participated actively. Frequency modulation stations also empowered the affected people by including their voices in programmes related to relief and reconstruction. The establishment of FM radio in such backward areas also caused an information revolution as it was not possible for the state-run radio to include the voices of such backward areas’ people in their programmes. And it was also difficult for the local people to approach state-run media. The use of local language, local content, awareness, educational programmes and providing the people an opportunity to take part in the decision-making processes and mobilising social change were the signs of development of a community. Similarly, the FM radio could play a very active role in the reconstruction process and the relief agencies could use FM stations to provide information regarding reconstruction. But, unfortunately the closure of the stations deprived the affected people from interesting information about the reconstruction. The role of FM stations as a watchdog was also very important and they effectively highlighted many issues of the affected people. The radio reports facilitated the victims and helped to resolve their problems. These reports also motivated the government and relief agencies who took prompt actions and removed the sense of deprivation of the affected people.
